# Supplementary material for: Differential expression of some termite neuropeptides and insulin/IGF-related hormones and their plausible functions in growth, reproduction and caste determination
Source: PeerJ. 2023 Apr 26;11:e15259. doi: 10.7717/peerj.15259 (PMC10148640; doi:10.7717/peerj.15259)
Supplement: Supplemental Information 1 [file peerj-11-15259-s001.pdf]

Supplementary Data

Differential expression of some termite neuropeptides and insulin/IGF-related hormones and their plausible functions in growth, reproduction and caste determination

Jan A. Veenstra

Table of contents

SRAs used	page 2
SRAs used for figure 5	page 4
Figure S1	page 5
Figure S2	page 6
Figure S3	page 7
Figure S4	page 8
Figure S5	page 9
Figure S6	page 10
Figure S7	page 11
Figure S8	page 12
Figure S9	page 13
Figure S10	page 14
Figure S11	page 15
Figure S12	page 16
Figure S13	page 17
Figure S14	page 18

SRAs used:

Coptotermes formosanus : SRR2155575, SRR2155576, SRR2155577, SRR2155578, SRR10273592, SRR10273593, SRR10273594, SRR10273595, SRR10273596 and SRR10273597.
Cryptocercus meridianus : SRR12393556, SRR12393557, SRR12393558, SRR12393559 and SRR12393572.
Cryptocercus punctulatus : DRR058700, DRR058701, DRR058702 and DRR058703.
Cryptocercus wrighti : SRR921587.
Cryptotermes secundus : ERR2615943, ERR2615944, ERR2615945, ERR2615946, ERR2615947, ERR2615948, ERR2615949, ERR2615952, ERR2615950, ERR2615951, ERR2615953, ERR2615954, SRR5457739, SRR5457740, SRR5457741, SRR5457742, SRR5457743, SRR5457744, SRR5457745, SRR5457746, SRR5457747, SRR5457748, SRR5457749, SRR12381713, SRR12381714, SRR12381715, SRR12381716, SRR12381717, SRR12381718, SRR12381719, SRR12381720, SRR12381721, SRR12381722, SRR12381723, SRR12381724, SRR12381725, SRR12381726, SRR12381727, SRR12381728, SRR12381729, SRR12381730, SRR12381731, SRR12381732, SRR12381733, SRR12381734, SRR12381735, SRR12381736, SRR12381737, SRR12381738, SRR12381739, SRR12381740, SRR12381741, SRR12381742, SRR12381743, SRR12381744, SRR12381745, SRR12381746, SRR12381747, SRR12381748, SRR12381749, SRR12381750, SRR12381751, SRR12381752, SRR12381753, SRR12381754, SRR12381755, SRR12381756, SRR13221217, SRR13221218, SRR13221219, SRR13221220, SRR13221221, SRR13221222, SRR13221223, SRR13221224, SRR13221225, SRR13221226, SRR13221227, SRR13221228, SRR13221229, SRR13221230, SRR13255030, SRR13255031, SRR13255032, SRR13255033, SRR13255034, SRR13255035, SRR13255036, SRR13255037, SRR13255038, SRR13255039, SRR13255040, SRR13255041, SRR13255042, SRR13255043, SRR13255044, SRR13255045, SRR13255046, SRR13255047, SRR13255048, SRR13255049, SRR13255050, SRR13255051, SRR13255052, SRR13255053, SRR13255054, SRR13426400, SRR13426401, SRR13426402, SRR13426403, SRR13426404, SRR13426405, SRR13426406, SRR13426407, SRR13426408, SRR13426409, SRR13426410, SRR13426411, SRR15145186, SRR15145187, SRR15145188, SRR15145189, SRR15145190, SRR15145191, SRR15145192, SRR15145193, SRR15145194, SRR15145195, SRR15145196, SRR15145197, SRR15145198, SRR15145199, SRR15145200, SRR15145201, SRR15145202, SRR15145203, SRR15145204, SRR15145205, SRR15145206, SRR15145207, SRR15145208, SRR15145209, SRR15145210, SRR15145211, SRR15145212, SRR15145213, SRR15145214, SRR15145215, SRR15145216 and SRR15145217.
Cryptotermes domesticus : SRR2039534.
Globitermes sulphureus : SRR7170939, SRR9968581 and SRR12393576.
Hodotermopsis sjostedti : DRR084180, DRR084181, DRR084182, DRR084183, DRR084184, DRR084185 and DRR084186.
Labritermes buttelreepeni : SRR9968587.
Macrotermes barneyi : SRR3178362, SRR3182796, SRR3182798, SRR3182799, SRR3182800, SRR3182801, SRR3182802, SRR3182803, SRR3182804, SRR3182805, SRR3182806, SRR3182808, SRR3182809, SRR3182810 and SRR3182811.
Macrotermes bellicosus : ERR2532157, ERR2532158, ERR2532159, ERR2532160, ERR2532161, ERR2532162, ERR2532163, ERR2532164, ERR2532165, ERR2532166, ERR2532167, ERR2532168, ERR2532169, ERR2532170, ERR2532171, ERR2532172, ERR2532173, ERR2532174, ERR2532175, ERR2532176, ERR2532177, ERR2532178, ERR2532179, ERR2532180, ERR2532181, ERR2532182, ERR2532183, ERR2532184, ERR2532185, ERR2532186, ERR2532187, ERR2532188, ERR2532189, ERR2532190, ERR2532191, ERR2532192, SRR14432658, SRR14432659, SRR14432660,

SRR14432661, SRR14432662, SRR14432663, SRR14432664, SRR14432665, SRR14432666, SRR14432667, SRR14432668, SRR14432669, SRR14432670 and SRR14432671.
Macrotermes natalensis : SRR789255, SRR789327, SRR789336, SRR789341, SRR789345, SRR789352, SRR789356, SRR789363, SRR789369, SRR789377, SRR5457275, SRR5457276, SRR5457277, SRR5457278, SRR5457279, SRR5457280, SRR5457281, SRR5457282, SRR5457283, SRR5507526, SRR5507527, SRR13396076, SRR13396077, SRR13396078, SRR13396079, SRR13396080, SRR13396081, SRR13396082, SRR13396083, SRR13396084, SRR13396085, SRR13396086, SRR13396087, SRR13396088, SRR13396089, SRR13396090, SRR13396091, SRR13396092, SRR13396093, SRR13396094, SRR13396095, SRR13396096, SRR13396097, SRR13396098, SRR13396099 and SRR13396100.
Mastotermes darwiniensis : SRR921616, SRR6869961, SRR8924830, SRR8924831 and SRR12393550.
Nasutitermes takasagoensis : DRR162556, DRR162557, DRR162558, DRR162559, DRR162560 and DRR162561.
Neotermes castaneus : SRR12393532, SRR12393533, SRR12393534, SRR12393535, SRR12393536, SRR12393537, SRR12393538, SRR12393539, SRR12393540, SRR12393541, SRR12393542, SRR12393543, SRR12393544, SRR12393545, SRR12393546, SRR12393547, SRR12393548, SRR12393549, SRR12393551, SRR12393560, SRR12393562, SRR12393563, SRR12393564, SRR12393565, SRR12393566, SRR12393567, SRR12393568, SRR12393569, SRR12393570, SRR12393571 and SRR12393573.
Prorhinotermes opinatus : SRR12393582.
Prorhinotermes simplex : SRR921637, SRR13236743, SRR13236744, SRR13236745, SRR13236746, SRR13236747, SRR13236748 and SRR13236749.
Reticulitermes aculabialis : SRR9140410, SRR9140411, SRR9140412, SRR9140413, SRR9140414, SRR9140415, SRR9140416, SRR9140417 and, SRR9140418.
Retitculitermes chinensis : SRR10604052, SRR10604053, SRR10604054, SRR10604055, SRR10604056, SRR10604057, SRR10604058, SRR10604059, SRR10604060, SRR10604061, SRR10604062, SRR10604063, SRR18067184, SRR18067185, SRR18067186, SRR18067187, SRR18067188 and SRR18067189.
Retitculitermes flavipes : SRR5341585, SRR5341586, SRR5341587, SRR5341588, SRR5341589, SRR5341590, SRR5341591, SRR5341592 and SRR5341593.
Retitculitermes labralis : SRR5801942, SRR5808263, SRR8707277, SRR8707278, SRR8707279, SRR9301201, SRR9301202, SRR9301203, SRR9301204, SRR9301205, SRR9301206, SRR9301207, SRR9301208 and SRR9301209.
Retitculitermes speratus : DRR030795, DRR030796, DRR030797, DRR030798, DRR030799, DRR030800, DRR030801, DRR030802, DRR030803, DRR030804, DRR030805, DRR030806, DRR030807, DRR030808, DRR030809, DRR030810, DRR030811, DRR030812, DRR030813, DRR030814, DRR030815, DRR030816, DRR030817, DRR030818, DRR030819, DRR030820, DRR030821, DRR030822, DRR030823, DRR030824, DRR030825, DRR030826, DRR030827, DRR030828, DRR030829, DRR030830, DRR030831, DRR030832, DRR030833, DRR030834, DRR030835, DRR030836, DRR030837, DRR030838, DRR030839, DRR030840, DRR030841, DRR030842, DRR030843, DRR030844, DRR030845, DRR030846, DRR030847, DRR030848, DRR030849, DRR030850, DRR030851, DRR030852, DRR030853, DRR030854, DRR090831, DRR090838, DRR090840, DRR090841, DRR090842, DRR090843, DRR090844, DRR090846, DRR090847, DRR090848, DRR090852, DRR090853, DRR090854, DRR090855, DRR090856, DRR090857, DRR090858, DRR090859, DRR090860, DRR090861, DRR090862, DRR090863, DRR090864, DRR090865, DRR252502, DRR252503, DRR252504, DRR252505, DRR266547,

DRR266548, DRR266549, DRR266550, DRR266551, DRR266552, DRR266553, DRR266554, DRR266555, DRR266556, DRR266557, DRR266558, DRR266559, DRR266560, DRR266561, DRR266562, DRR332717, DRR332718, DRR332719, DRR332720, DRR332721, DRR332722, DRR332723, DRR332724, DRR332725, DRR332726, DRR332727, DRR332728, DRR332729, DRR332730, DRR332731, DRR332732, DRR332733, DRR332734, DRR332735, DRR332736, DRR332737, DRR332738, DRR332739, DRR332740, DRR332741, DRR332742, DRR332743, DRR332744, DRR332745, DRR332746, DRR332747, DRR332748, DRR332749, DRR332750, DRR332751, DRR332752, DRR332753, DRR332754, DRR332755, DRR332756, DRR332757, DRR332758, DRR332759, DRR332760, DRR332761, DRR332762, DRR332763, DRR332764, DRR332765, DRR332766, DRR332767, DRR332768, DRR332769, DRR332770, DRR332771, DRR332772, DRR332773, DRR332774, DRR332775, DRR332776, DRR332777, DRR332778, DRR332779, DRR332780, DRR332781, DRR332782, DRR332783, DRR332784, DRR332785, DRR332786, DRR332787, DRR332788, DRR357004, DRR357005, DRR357006, DRR357007, DRR357008, DRR357009, DRR357010, DRR357011, DRR357012, DRR357013, DRR357014, DRR357015, DRR357016, DRR357017, DRR357018 and DRR357019.
Zootermopsis nevadensis : DRR110536, DRR110537, DRR110538, DRR110539, DRR110540, DRR110541, DRR110542, DRR110543, DRR110544, DRR110545, DRR110546, DRR110547, DRR110548, DRR110549, DRR110550, DRR139981, DRR139982, DRR139983, DRR139984, DRR139985, DRR139986, DRR139987, DRR139988, DRR151559, DRR151560, DRR151561, DRR151562, DRR151563, DRR151564, DRR151565, DRR151566, DRR151567, DRR151568, DRR151569, DRR151570, SRR863596, SRR863597, SRR863598, SRR863599, SRR863601, SRR863602, SRR863603, SRR863604, SRR863605, SRR863606, SRR863612, SRR863613, SRR1167035, SRR1167037, SRR1167039, SRR1167040, SRR1167041, SRR1167042, SRR1167043, SRR1167044, SRR1167178, SRR1167247, SRR1167255, SRR1167256, SRR3139733, SRR3139734, SRR3139735, SRR3139736, SRR3139737, SRR3139738, SRR3139739, SRR3139740, SRR3139741, SRR3139742 and SRR3139743.

SRAs used for figure 5:
ERR2615943, ERR2615944, ERR2615945, ERR2615946, SRR5457742, SRR5457743, SRR5457744, SRR5457745, SRR12381714, SRR12381716, SRR12381718, SRR12381721, SRR12381723, SRR12381725, SRR12381727, SRR12381728, SRR12381730, SRR12381732, SRR12381734, SRR12381736, SRR12381738, SRR12381740, SRR12381743, SRR12381744, SRR12381745, SRR12381746, SRR12381747, SRR12381748, SRR12381755 and SRR12381756.

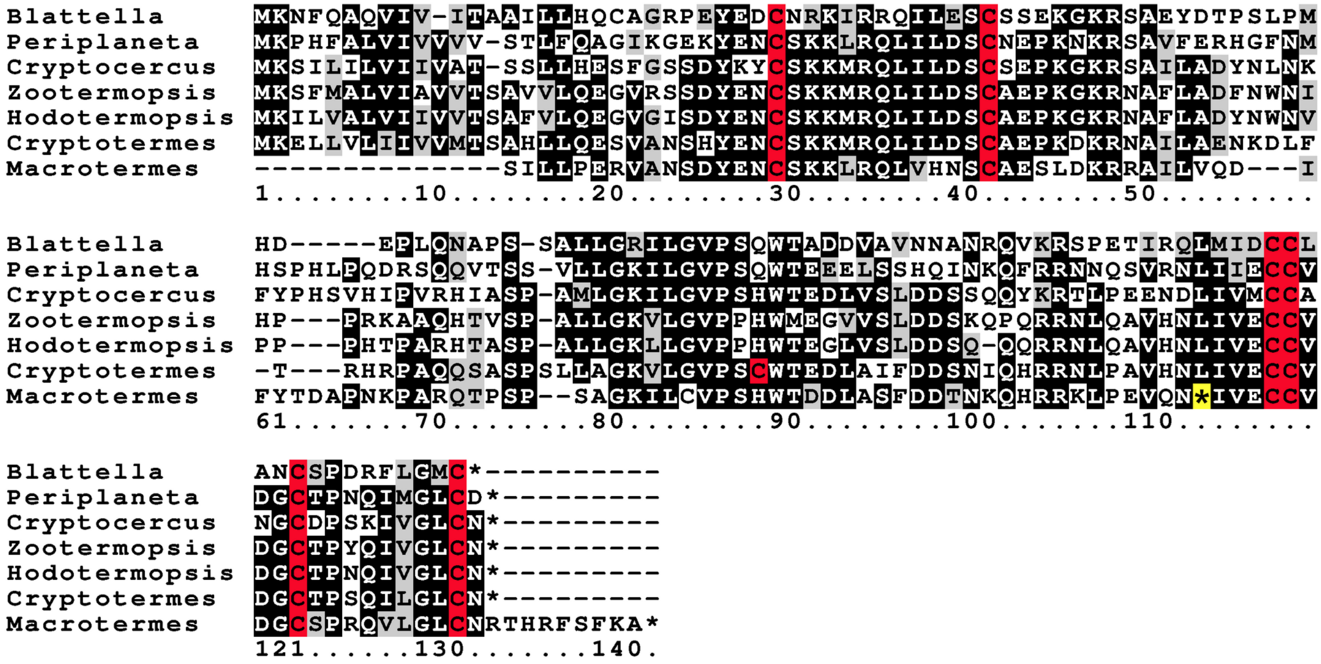

Figure S1. Sequence comparison of predicted gonadulin sequences from three cockroach species, *Blattella germanica*, *Periplaneta americana* and *Cryptocercus wrighti* and four termite species, *Zootermopis nevadensis, Hodotermopsis sjostedti*, *Cryptotermes secundus* and *Macrotermes natalensis.* Note that the *Macrotermes* sequence is derived from a pseudogene as it has an inframe stop codon and appears to lack the first part of the signal peptide. Also note the unusual seventh cysteine in the *Cryptotermes* sequence. Identical amino acid residues are highlighted in black, conservative substitutions are in grey and the cysteine residues are highlighted in red. An inframe stop codon in the *Macrotermes* sequence is highlighted in yellow.

```
Blattella       MLKCGIVTALVLVTTMVSGAPTIRMQMCGSQLANTLAQICSAYGYHDPFSQTRRVNSPSS
Periplaneta     ----MS-LLFLVLLASATASPLGRMQLCGSELANKLAEICAVYGYNDPFRHAHY-ED-ST
Zootermopsis    -MPLLSVVRIFLLTMTVSASPLGRLQLCGSELANKLAEICSIQGYNDPFRHSMYYEV-SP
Hodotermopsis   -MSLLSVVRIFLLTMTVTASPLRRMQLCGSELANKLAEICSIQGYNDPFRQSMYYEI-SP
Cryptotermes    MPLALSLVRVFLLTMTVAASPLGRLQLCGSELANKLAEICSVQGYNDPFRNSMYYEI-SP
Coptotermes     MSLHLCVVRMLLLTMTVAASPLGMLQLCGSALANKLAEICAVQGYNDPFKHSMYYDM-SP
Prorhinotermes  MSLHLCVVRILLVTMTVASSPLGKLQLCGSDLANKLAEICAAQGYNDPFKHSMYYEM-SP
Reticulitermes  MSLHLCVVRMLLLTMTVAASPLGMLQLCGSALANKLAEICAAQGYNDPFKHSMYYEM-SP
Macrotermes     MSPHLSVVRILLLTVTVAASPLGRLQLCGSALANKLAEICSVQGYNDPFRHSMYYEM-SS
Labritermes     MSPHLSVARVLLLTVAVAASPLGRLQLCGSDLANKLAEICAAQGYNDPFRHSMYYEL-SP
Nasutitermes    -----------------------------------------VQGYNDPFRHNMYYEM-SP
                1........10........20........30........40........50.........

Blattella       GVNTTPNRLRVRRGVADECCKTGCTLDTMEQYCSAPLTPAQRARFLQQYQSNALNRILQE
Periplaneta     YDSITPTRTRVKRGVADECCKTGCSQETLEQYCNPPLKSPDRSKVLKISEDHSINHIPQD
Zootermopsis    FEGGVSSTTRSKRGVADECCKTGCSWDTLEQYCNPPLSTPDRPHNLQETEDHSINHIAQV
Hodotermopsis   FGGVVPLTTRSKRGVADECCKTGCSWDTLEQYCNPPLSTPDRPHNLQETEDHSTNHISQD
Cryptotermes    LDNGVPLMTRSKRGVADECCKTGCSWDTLELYCNPPLPTAQRPHNLQEAEDHSMNHIPLD
Coptotermes     FENGIPITTRSKRGVADECCKKGCSWETLEQYCNPPLPTPSRTDNLQATEEHSMNRIPHD
Prorhinotermes  FENGIPMTTRSKRGVADECCKKGCSWDTLEQYCNPPLRTPNRTDNLQATEEHSXX-----
Reticulitermes  LDNGIPVTTRSKRGVADECCKKGCSWDTLEQYCNPPLPAPNRTDNLQATEEHSMNRIPQD
Macrotermes     LENGIPMTTRSKRGVADECCKKGCSWDALEQYCNPPNPTRNRTDNLQATEEHSMNRIPQD
Labritermes     LENSIPMTTRGKRGVADECCKKGCSWDTLEQYCNPPIPTPNRTDNLQSTEEQSINRIPHD
Nasutitermes    LENGIPMTTRAKRGVADECCKKGCSWNTLEQYCNPPIPSSNRTDILQETEEQSMNRIPHD
                61.......70........80........90........100.......110........

Blattella       VPSGSSLN----AGSKISKDPKNDLSSKVRRQQDKKGQRGNNRCRCRRRRRRGKGDSEEI
Periplaneta     DSAASAVLTSEVRRSSIAREEKNDLVSKVRGHHDKKGRRANNRCRCRRRRRRGKGDLEEL
Zootermopsis    DSEGSAG-----QPTTITREQKNDLVSKVRSHHEKKGRRGNNRCRCRRRRRRGKGDIEDP
Hodotermopsis   VSEGSAG-----LPPATTREQKNDVVSKVRSHHDRKGRRGNNRCRCRRRRRRGKGDIEDP
Cryptotermes    DSTGSEA-----RPPAKSRAQKNDLVSKVRSHHDKKGRRGNNRCRCRRRRRRGKGDMEDA
Coptotermes     DSAGSAG-----RLPLRARDQKSDLISKVRSHHEKKGRRGNNRCRCRRRRRRGKGDIEDP
Prorhinotermes  ---------------------------XXRSHHEKKGRRGNNRCRCRRRRRRGKGDTEDP
Reticulitermes  DSAGSAA-----RPPIMTRDQKSDLISKVRSHHEKRGRRGNNRCRCRRRRRRGKGDIEDP
Macrotermes     DSAGTAG-----RPSIMTRDQKSDLISKVRSHHEKKGRRGNNRCRCRRRRRRGKGDIEDP
Labritermes     DSASSAA-----RPQIMTRDQKSDLISKVRSHNEKKGRRANNRCRCRRRRRRGKGDIEDP
Nasutitermes    DSASSAA-----RAPITTRDQKSDLISKVRSHHEKKGRRGNNRCRCRRRRRRGKG-----
                121......130.......140.......150.......160.......170........

Blattella       ERQQNHIAPVIGTINPSYFGVPVFLSPRLKKQETQQDRHRK*-
Periplaneta     ERHLNKIAPVIGTINPSYLGIPVILPPRIRKEEMSLQDYGTK*
Zootermopsis    EILENKIAPVIGTINPAYFANPVILPPRIRKEEMALQDTK*--
Hodotermopsis   EIFENKIAPVIGTINPSYFANPVILPSRIRKEEMSLQDTK*--
Cryptotermes    EILENKIAPVIGTINPSYFANPVILPPRVRKEEMSLQDRK*--
Coptotermes     EVLENKIAPVIGTINPSYFANPVILSQRIRKEETSLQETK*--
Prorhinotermes  EVLENKIAPVIGTINPSYFGNPVILPPRIRKEEMSLQEKK*--
Reticulitermes  EVLENKIAPVIGTINPSYFANPVILPPRIRKEEMSLQETK*--
Macrotermes     EVLENKIAPVIGTINPSYFANPVILPPRIRKEEMSLQETQ*--
Labritermes     EVLENKIAPVIGTINPSYFANPVILPPRIRKEGMSLQETQ*--
Nasutitermes    -------------------------------------------
                181......190.......200.......210.......220.
```

Figure S2. Sequence alignment of the predicted long IGFs from two cockroaches, *Blattella germanica* and *Periplaneta americana*, and nine termite species, *Zootermopis nevadensis, Hodotermopsis sjostedti, Cryptotermes secundus, Coptotermes formosanus, Prorhinotermes simplex, Reticulitermes speratus, Macrotermes natalensis, Labritermes buttelreepeni* and *Nasutitermes takasagoensis.* Note that the *Prorhinotermes* and *Nasutitermes* sequences are incomplete due to lack of data. Highlighting of residues as in figure S1.

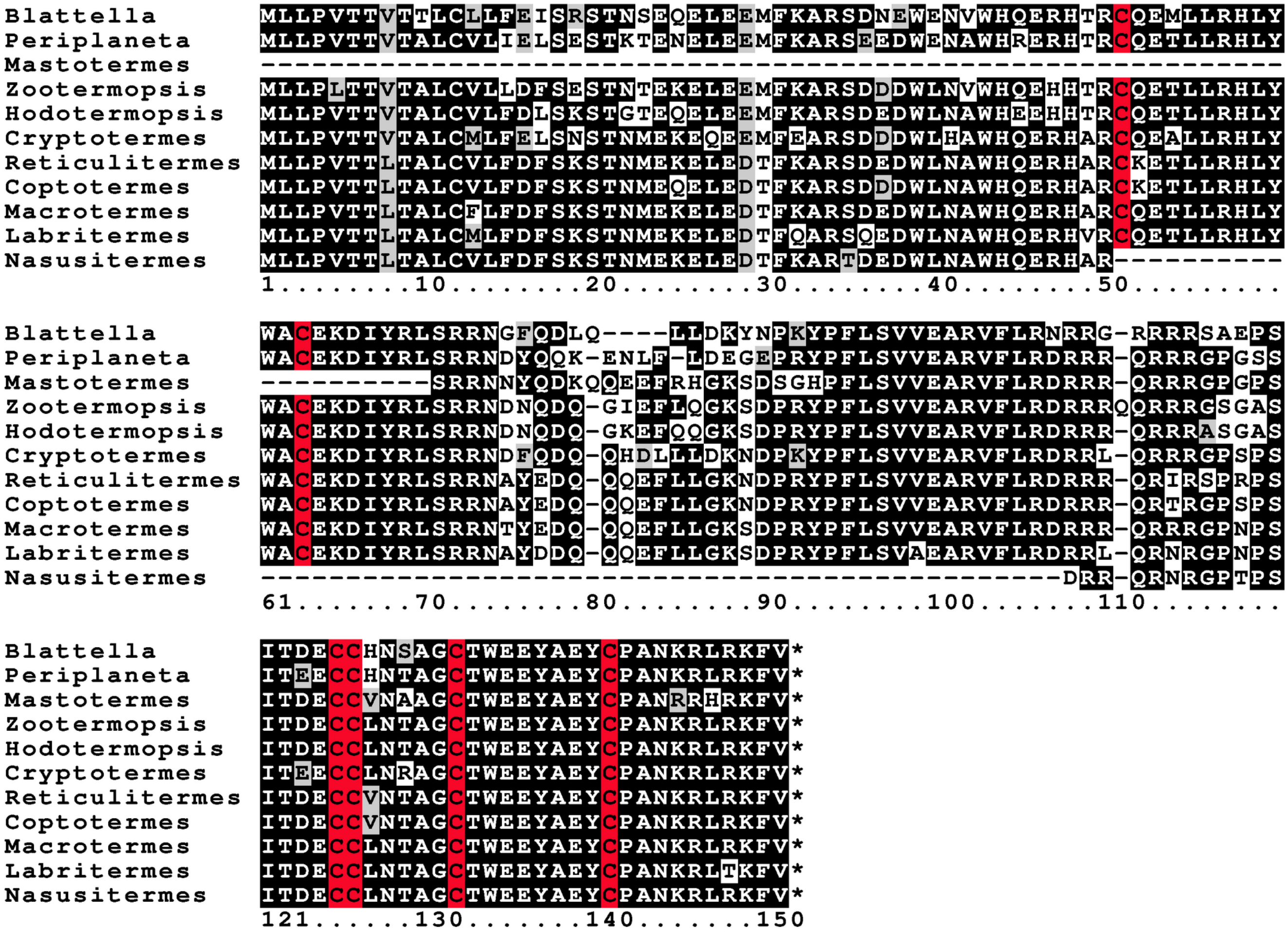

Figure S3. Sequence alignment of the predicted dilp7 orthologs from two cockroaches, *Blattella germanica* and *Periplaneta americana*, and nine termite species, *Mastotermes darwiniensis, Zootermopis nevadensis, Hodotermopsis sjostedti, Cryptotermes secundus, Coptotermes formosanus, Reticulitermes speratus, Macrotermes natalensis, Labritermes buttelreepeni* and *Nasutitermes takasagoensis.* Note that the *Mastotermes* and *Nasutitermes* sequences are incomplete due to lack of data. Highlighting of residues as in figure S1.

```
Blattella        --MWRICLQLVAIAALCLCTLAQAQSDLFQFADKRNTNKYCGRNLANMLQLVCNGNYYPM
Periplaneta      --MWRLCLRLVAVAALCLCTLAQAQSDLFQFADKRNTNKYCGRNLANMLQLVCNGNYYPM
Cryptocercus     --MWRLCLRLVAVAALCLCTLAQAQSDLFQFSDKRSTNKYCGRNLANMLQFVCNGNYYPM
Mastotermes      --MWRLCLRLVAIAAVCLCTLAQAQSDLFQLGDKRNTNKYCGRNLANMLRYVCNGNYYPM
Hodotermopsis    --MCRLYLRLVAIAALCLCTLAQAQSDLFQLGDKRNTNKYCGRNLANMLRYVCNGNYYPM
Zootermopsis     --MWRLYLRLVAIAALCLCTLAQAQSDLFQLGDKRNTNKYCGRNLANMLRYVCNGNYYPM
Cryptotermes     --MWRLCLQLVAIAALCLCTLAEAQSGLFQLADKRYTNKYCGRKLSNMLQFVCNGNYYPM
Prorhinotermes   MKMWRLCLQMVAIAALCLCTLAQAQSDLFQLSDKRITNKYCGRNLANMLRYVCNGNYYPM
Reticulitermes   MKMWRLCLQLVAIAALCLRTLAQAQSDLFQLSDKINTNKYCGRNLANMLRYVCNGNYYAR
Coptotermes      --MWRLCLQLLAIAALCLCTLAQAQSDLFQLSDKRNTNKYCGRNLANMLQYVCNGNYYPM
Macroteremes     --MWRHCLQLVAIAALCLHTLAQFQSDLFQLSDKPNTNKYCGRNLANMLQYVCNGKYYPM
Labritermes      --MWMFCLQLVAISALCLHALAQLQSDYFQLSDKRNTNKYCGRNLANMLQYVCNGKYYPM
Nasutitermes     MKMWRLCLRLVAIAALCLHALAQVQSDLFQLSDKRNTNKYCGRNLANMLQYVCNGKYYPM
                 1.......10........20........30........40........50.........

Blattella        FKKSSQDMDDMNDSGFWIQPSTMEEQQLQYPFRSRSSASALVSGSFRRRTRGVYDECCRK
Periplaneta      FKKSSQDVDDMNDSGFWIQSQPVQEPQLQFPFRSRTSAS-LIPDSFRRRTRGVYDECCRK
Cryptocercus     FKKASQDTEDMNDSGFWLQPPPVEEPELQFPFRSRSNAASLVPGHFRRHTRGVYDECCRK
Mastotermes      FKKASQDVEDTNDSGIWILSPPTEEPQLQFPFRSRSNAASLVPGSFRRHTRGVYDECCRK
Hodotermopsis    FKKASQDVEDVNDSGIWIQPLPVEEPQLQFPFRSRSNAASLVPGSTRRHTRGVYDECCRK
Zootermopsis     FKKASQDVEDVNDSGIWIQPLPIEEPQLQYPFHSRSNAATLVPGSLRRHTRGVYDECCRK
Cryptotermes     FKKTSQDVEDMNDSDFWIQTTPIDEQEVQFPFRSRSKAVTAVPGSFRRHTRGVYDECCRK
Prorhinotermes   FKKATQDTEDMNDSASWIQPPSIEEPESQFPFRSRSNAATLVPGSFRRHTRGVYDECCRK
Reticulitermes   FKKATQDVEDKNDSAFWIQPPHTE--ELQFPFRSRSDAATLVPGSFRRYTRGVYDECCRK
Coptotermes      FKKATQDVEDMNDSAFWIQPSPIEEPESQFPFRSRSNAATLVPGSFRRHTRGVYDECCRK
Macroteremes     FKKATQDAEDMNDSAFWIQPPPIDEPEIQFPFRPRSNAATLVSGSFRRHTRGVYDECCRK
Labritermes      FKKATQDAEDMNDSVFWIQPPPVEEPEFRFPFRPRANAATLVSGSFRRNTRGVYDECCRK
Nasutitermes     FKKATQDAEDMNDSAFWMQPPPTEEPEIQFPFRPRSDAATFVSGSFRRYTRGVYDECCRK
                 61.......70........80........90........100.......110........

Blattella        SCSIQEMASYCGKR*
Periplaneta      SCTVQEMASYCGRR*
Cryptocercus     SCSIQEMASYCGKR*
Mastotermes      SCTIQEMASYCGRR*
Hodotermopsis    SCTIQEMVSYCGRR*
Zootermopsis     SCTIQEMVSYCGSR*
Cryptotermes     SCTIQEMASYCGR*-
Prorhinotermes   SCTVQEIASYCARR*
Reticulitermes   SCTIQEIASYCGRR*
Coptotermes      SCSIQEIASYCGRR*
Macroteremes     SCTIQEIASYCGRR*
Labritermes      SCTIQEIASYCGRR*
Nasutitermes     SCTIQEIASYCGRR*
                 121.......130...
```

Figure S4. Sequence alignment of the predicted atripins from three cockroach species, *Blattella germanica*, *Periplaneta americana* and *Cryptocercus wrighti* and ten termite species, *Mastotermes darwiniensis*, *Zootermopis nevadensis*, *Hodotermopsis sjostedti*, *Cryptotermes secundus*, *Prorhinotermes simplex*, *Coptotermes formosanus*, *Reticulitermes speratus*, *Macrotermes natalensis*, *Labritermes buttelreepeni* and *Nasutitermes takasagoensis*. Highlighting of residues as in figure S1.

```
Periplaneta-3    ----MWRV-VMVAACLCVLCESQSDLFQPQDKR-GSRRYCGPTLVSTLHIICNGTYYTNI
Periplaneta-4    ----MLKVAVLMAACLCVLCESQSDML---EKRDSARRYCGRNLADVMHIVCNGIYNGNP
Cryptocercus     MWRSCLRALVVVALCLCSMSESQSDVFQFQDKRAEAKRYCGNNLVAALQFLCDGRYYSNF
Mastotermes      MWRVCFRVMVVVAMCLCSLPQSQSDVLQFPDKRQEAKRYCGDNLVRILQFLCDGIYYSNT
Hodotermopsis    MWRACFRVMVVVAMCLCSLAQSQSDIFQFPDKRPETKRYCGSNLVDILQLLCNGKYYSNT
Zootermopsis     MWRACFRIVVVVALCLCSLAQSQSDIFPFPDKRPETKRYCGSNLVDILQLVCNGKYYSNI
Cryptotermes     MWRACFTVMVVVAMCLCSLADSQSDLYQFPSKRPDSKRYCGSNLVKILQFLCDGYYNANI
Prorhinotermes   MWKACFRMIVFVAMCLCPLAESQSDIFQFPDKRLEARRYCGNNLVDKLQLLCNGYYYSNI
Reticulitermes   MWRVCFRMMVFAAMCLCSLAESQSDIFQFPDKRLETKRYCGNNLVDILQFLCNGYYYSNI
Coptotermes      MWRVCFRMTVFAAMCLCSLAESQSDIFQFPDKRPETKRYCGNNLVDILQLLCNGYYYSNI
Macroteremes     MWRACFRMMVFVAMCLCSLAESQSDIFQFPDKRLESKRYCGSNLVDMLQFLCNGYYYSNI
Labritermes      MWRVCFRTTVFVAVCLCSLAESQSDIFQFPDKRLENKRYCGNNLVDILQFLCNGYYYSNI
Nasutitermes     MWRSCFRMMVLVAMCLCSLAGSQSDIFQFPDKRLENKRYCGNNLVDILQFVCNGYYYSNI
                 1.......10........20........30........40........50.........

Periplaneta-3    RPL-----------SRAQKKAWPDSDDDVWMELQLPENAERYPFRSRASAVAFPRRVFKR-
Periplaneta-4    R-------------AATQKKSWPDADDDLW----PVEEQADFPFRSRASA----HRILKRQ
Cryptocercus     NTN---NSNNFNQHAGQKKSVSEMDDDYWLKLQPVEEHLKFPFRSR----TFAHKIFKR-
Mastotermes      NLNNNN-----NQRVGRKKSVPEVDEDFWLQLQPVEEQMKFPFLSRSSASTFAHRIFKR-
Hodotermopsis    NSNN--SNNNYNPHVGRKKSMPEVEEDFWQQFQPVEEQMKFPFRSRSSSSAFAHRIFKR-
Zootermopsis     NN------SNNYSPHVGRKKSMPEADEDFWQQLQPVEEQMKFPFRSRSSVSTFAHRIFKR-
Cryptotermes     S------PNNYGTRVGRKKSIPEADDDLWLQLQPAEEEIKFPFRPRSSASIFSHRMFKR-
Prorhinotermes   NANNNNNNNNYSPHAGRKKSMPEVDEDLWHQLQPVEEHMTFPFRSRSSASTFLHRIFKR-
Reticulitermes   NSN---NSNNYSPHTGRKKSMPEVDEDFWHQLQPLEEHMKFPFRSRSSASTFLHRIFKR-
Coptotermes      NSN---TSNNYSPHAGRKKSMPEVDEDFWHELQPAEEHMKFPFRSRSSASTFLHRIFKR-
Macroteremes     NSN---NSNNYSPHAGRKKSMPEVDEDLWHELEPVAEHIKFPFRSRSSASTFLHRIFKR-
Labritermes      NSS---NSNNYSPHAGRKKSVPEADEDLWHELEPVEEHMKFPFRSRSSASTFLHRIFKR-
Nasutitermes     NSN---NSYNYSPHAGRKKSVPEVDEDLWREIEPVEEHLKFPFRSRSSASTFLHRIFKR-
                 61.......70........80........90........100.......110........

Periplaneta-3    QSWGVADECCVRKGCDYNELSSYCAP*----
Periplaneta-4    SPIGIAYECCINKGCTIHELRTYCGR*----
Cryptocercus     HPGGVAYECCIIKGCTINELMSYCMPSTEQ*
Mastotermes      HPVGVAYECCISKGCTVQELSSYCGRS*---
Hodotermopsis    HTVGVAYECCINKGCTMHELRSYCAP*----
Zootermopsis     HTVGVAYECCINKGCTVYELRSYCAP*----
Cryptotermes     HSVGVAYECCINKGCTVDELKSYCGR*----
Prorhinotermes   HAVGVAYECCISKGCTIHELRGYCAP*----
Reticulitermes   HAVGVAYECCISKGCTIHELRGYCGP*----
Coptotermes      HAVGVAYECCISKGCTIHELRSYCGP*----
Macroteremes     HAVGVAYECCISKGCTIPELRSYCGS*----
Labritermes      HAVGVAYECCISKGCTIHELRSYCAP*----
Nasutitermes     HAVGVAYECCISKGCTIHELRSYCGP*----
                 121......130.......140.........
```

Figure S5. Sequence alignment of the predicted birpins from two cockroach species, *Periplaneta americana* and *Cryptocercus wrighti* and ten termite species, *Mastotermes darwiniensis, Zootermopis nevadensis, Hodotermopsis sjostedti, Cryptotermes secundus, Prorhinotermes simplex, Coptotermes formosanus, Reticulitermes speratus, Macrotermes natalensis, Labritermes buttelreepeni* and *Nasutitermes takasagoensis.* Note that these sequences are not as well conserved as the atirpin sequences (Fig. S4). Highlighting of residues as in figure S1.

```
Cryptocercus      -MKNLFLRATVIGMICTCVLPDRQINPSYLLRKREAVHRYCGPILANVLRFICNGSYHTE
Mastotermes       -MRNILMRVTVIGMICSWALPESHLS----LRKREAAFRYCGPNLANILRLLCNGSYHTD
Hodotermopsis     -MRNILLRVTLIGMICSLVLPDSQLS----VRKRETEYRYCGPNLANILRFICNGSYHTD
Zootermopsis      -MRTILLRLTVIGMICSWALPDSQLG----VRKRETAYRYCGPNLANILRLICNGSYHTD
Cryptotermes      -MRNIFLRVTVFAMICSWALPESQLG----MRKREAAYRYCGPNLANVLRFICNGSYHTD
Prorhinotermes    MMRNIFLRLTVIGMLCSWALPDSQLG----VRKRETAYRYCGSNLANILRMLCNGSYHTD
Reticulitermes    -MRNIFLRLAVIGMICSWALPDSQLG----VRKRETAIRYCGPRLANILRLLCNGSYHTD
Coptotermes       -MRNVFLRLAVIGMICSWALPDSQLG----VRKRESAYRYCGPNLANILRFLCNGSYHTD
Macroteremes      -MRSIFLRVAVIGMICSWALPDSQLG----VRKRETAHRYCGSNLANILRFICNGSYNTN
Labritermes       -MRNIFLRLAVIGMICSWALTDSQLG----MRKRETSYRYCGHYLSNILNFICNGSYYD-
Nasutitermes      -MTNIFLRLAVIGMICSWALPDSQLG----VRKRETSRRYCGPYLPNILRLICNGTYHTY
                  1.......10........20........30........40........50.........

Cryptocercus      EHWKRNGGGSAIQWKP-------------SQDVDDSLSIQPEGAEDAFPFQPRWIASQLT
Mastotermes       EHWKRNVATIQKKQAP---------------DMDGSLWIQPAAAEEAFPFRPWWMANQLT
Hodotermopsis     EHWKRNAGTIQKKHEQ---------------DMGLDLWTRPGAAAEQFPFRPWWMANQLT
Zootermopsis      EDWKRNGGSIQKKQEH---------------DKELDFWTPPEAAMEQFPFRTWWMANQFS
Cryptotermes      EHWKRNSGSTEMNKAQ---------------DTDSSPWFQTGDAEEVFPFRPRWMANQFT
Prorhinotermes    DHWKRNGGSVQMKQAQ---------------DIDGSLWVQPGDS-EDFPFRPWWMASQLT
Reticulitermes    DHYKRNSGSIQAKQAQ---------------DMDDSLWIQPGDS-EQFPFQPWWMANQLT
Coptotermes       DHYKRNAGSVQVKQAQ---------------DVDGSLWTQPEDS-EHFPFRPWWMANQLT
Macroteremes      DYWKRNTGSIQVKQAQ---------------DVDDSLWIQPEDS-DQFPFRPWWVANQLT
Labritermes       EPWKRNAGSVQGKQAQ---------------DVQGNPWIQPGDS-EQFPFRPWWIANQLK
Nasutitermes      D-WKRNGGSTQVKQAQEKPEIGENGVQRDESDVDGSLWGQPGDS-EQFPFRPWWTANQLT
                  61......70........80........90........100.......110........

Cryptocercus      NKVFRRQRRNVGIVDECCIFKGCTISELSGYCRNREATTT*---
Mastotermes       NRVFRRQTRDVGIVEECCMFKGCTLNELTEYCADR*--------
Hodotermopsis     NRVFRRQRKDAGIVEECCVFKGCTFSELSEYCADR*--------
Zootermopsis      NRVFRRQRRDAGIVEECCVFKGCTISELSEYCAES*--------
Cryptotermes      NRNFRRQRRSAGIVEECCVFKGCTVSELSEYCSAR*--------
Prorhinotermes    NRVFRRQRRNVGIVDECCVFKGCTVSELTEYCSDRAVSRPVTI*
Reticulitermes    NRVFRRQRRNVGIVEECCVFKGCTVSELSEYCADRVVSGPVTI*
Coptotermes       NRVFRRQRRNVGIVEECCIFKGCTINELSEYCAERVVSPPVTM*
Macroteremes      NRVFRRQRRNVGIVEECCIFKGCTRTELSEYCAERVVSEPVAM*
Labritermes       KRVFRRQRRR--IVEECCV-KGCNYSELSTYCSATVVSETETM*
Nasutitermes      NRVFRRQRRNVGIVEECCVFKGCTYSELSEYCADRVFSGPVTM*
                  121.....130.......140.......150.......160..
```

Figure S6. Sequence alignment of the predicted cirpins from one cockroach species, *Cryptocercus wrighti* and ten termite species, *Mastotermes darwiniensis, Zootermopis nevadensis, Hodotermopsis sjostedti, Cryptotermes secundus, Prorhinotermes simplex, Coptotermes formosanus, Reticulitermes speratus, Macrotermes natalensis, Labritermes buttelreepeni* and *Nasutitermes takasagoensis.* Note that these sequences are not as well conserved as the atirpin sequences (Fig. S4). Highlighting of residues as in figure S1.

```
Periplaneta-2       MWKLCLLVM----VTCACALPEN-------LNKRDSSHRYCGRNLVNILSFVCSGRGYNG
Cryptocercus        MWKLCLRVA-LIGLICVCALPENPSTMLQLVSKRETTHRYCGKILVNTLRFLCRSQ-YNG
Mastotermes         MRRLCLHVM-LIGVICVCALPENPSAVLQLVRKRDATHRYCGSNLVTTLRFLCRSQ-YNG
Hodotermopsis       MWRLCSRVMMLIGIICVCALPDHPSTAAKIVQKRQTTHRYCGKNLVDVLKFLCRGQ-YNG
Zootermopsis        MWRLCLRAM-LISMICVCALTDNPSTTPKFVEKR-EAHRYCGRNLANILKFLCQGH-YNG
Cryptotermes        MWRLFLRVM-LIGVICACALPENPSFI-KSVRKRDTSQRYCGKNLVNILRFLCRSQ-YNG
Prorhinotermes      MWRLCLPVM-LIGAICVCSLPGDPSSMSKLVTKREAEQRYCGTKLAAILRYVCNGT-YFA
Reticulitermes-1    MWRLSLRVM-LIAVICVCVLSGIPSSMSKYVNKREKEQRYCGSKLADKLREVCNGI-YES
Reticulitermes-2    MARLWLRVM-LIAVIGVCAVFEKPSSMSKYVKKRETEERYCGSKLADKLREVC-------
Reticulitermes-3    MWRLCLRVM-LIGVICVCALPENPSLMSKFVKKRETQQRFCGSKLVAILRLLCQGK-YHG
Coptotermes-1       MWRLCLSVV-LIGVICVCALPENPSLMSKLVQKRETQQRLCGNNLVAILRLLCQSK-YHG
Coptotermes-2       MWRLWLRVM-LITAICACAVFEKPSSMSKSVKKRQTEERYCGSKLADKLMEVCNGT-YEG
Coptotermes-3       MWTLSLRVM-LTAVICVRVLSGIPSSMSRFVQKREIEQRYCGSKLYYKLREVCNST-YQG
Coptotermes-4       MWRQWLRVM-LITAICACAVFEKPSSMSKFVKKRQTEV*YCGSKLADKLMEVCKST-YEG
Macroteremes-1      MWRLCLRVM-LIAVICVCALPEKPSVMSKLVRKRETQQRYCGSRLVNILKFLCQSK-YYG
Macroteremes-2      MWRLCLRVM-LIAVICVCALPDKLSVMSKFLKKRETEQRYCGSRLVDKLKEVCNST-YHS
Labritermes-1       MWRLCLRVM-LVAVICVCALPAKPSVMSKLVRKRETQQRYCGSRLVHILKFLCQSK-YYG
Labritermes-2       MWKLFLRVM-LVAVICVCALPAKPSVMSKLVRKRETQQRYCGSRLVHILKFLCQSK-YYG
                    1........10........20........30........40........50........

Periplaneta-2       VEEMEERKRN--------------------------SHALPDADDAFWWLQLS---DEEL
Cryptocercus        IEDETKRNADPIQK---------------------KSLQ--DTDEYLWIQPQAVDERQEI
Mastotermes         IDDERKRNAEPIVK---------------------KSLQVSDPDNSLWTQLQTVDELPET
Hodotermopsis       MEYERKKRDV-YYS---------------------EEDPFEDRDDDFWSQSEDLSGFPNL
Zootermopsis        IDYDRKRRDVDFYG---------------------EPKLYGDHDYDFWSQAEDVDDFADL
Cryptotermes        IEEERKRSEETSQR---------------------KVAR--DLEYVQWSRPKPVE-EPEI
Prorhinotermes      PEAKRKRDT-----------------------GFFENMQEEDSDTDLWMESQDVEEFQDA
Reticulitermes-1    MDKKMK----------------------------------RDLDTLQWIQSHAEEVVSNT
Reticulitermes-2    ----------------------------------------SDLDTVLWFQSQAEKVVSNT
Reticulitermes-3    MFERRKRDT-----------------------DFFLWRSPQDLDTLPWIQSHAEEVVSNT
Coptotermes-1       MFERRKRDT-----------------------HMFHSRSPQDLDTLQWIQSQAEEEVPKT
Coptotermes-2       PNNTDQGVNNTDLGVNNTDKGVNNTDLGVNTDQGVNTPRKRDLRTVLWFQSQAEKEVRNT
Coptotermes-3       MENKHK----------------------------------RDIHTVLWFQSRGEKEVPNT
Coptotermes-4       PNNTDQ------------------------------------------------------
Macroteremes-1      MEERRRRDT-----------------------EFFHTRSPQDMGTVLWNQSQAEEQVPNT
Macroteremes-2      MEMK-----------------------------------KRDMGTVLWNQSQVEEQVPNT
Labritermes-1       MEERRKRDT-----------------------EFFHRRSPQDMGTVLWNQSPAEEQVPNP
Labritermes-2       MEERRKRDT-----------------------EFFHRRSPQDLDNVLWNQTQTEENVPNP
                    61.......70........80........90........100.......110........

Periplaneta-2       QFPFRSRAAANTLSNREFRRQ-TRNDRGIVEECCIVKGCTINELADYCLSR*--------
Cryptocercus        QFPFRPRSIANTFANRIFRRQ-ERSDGGIVDECCIYKGCTLNELTAYCSNG*--------
Mastotermes         EFPFRSRSIANTFANRVFRRR-ERSDGGIVDECCIYKGCTVNELTEYCSSR*--------
Hodotermopsis       QYPFRSRPAANTLAN-MFRRRQK-RGDGIVEECCVSKGCTVNELRDYCSPR*--------
Zootermopsis        HYPFRPRSVSSVLLNKLFRRRPKRNNGGIVEECCIYKGCTLSELTEYCSPVQ*-------
Cryptotermes        RFPFRSRAAANT-----FRRQ-KRNDGGIVDECCIYKGCTVNELTDYCSSRR*-------
Prorhinotermes      KFPFNSHSEASTFKNRRFRRE-DGNYTGIVDECCVYKGCTLTEVAEYCAPQQPDASTTNS
Reticulitermes-1    KFPFRPRSIANKFKKRSLRRQ-KRTSGGVADECCINKGCTIGELREYCAER*--------
Reticulitermes-2    KFPFQPRSVVNKFKKRSLRRQ-KRDESGVAYECCINKGCTIGELRDYCAER*--------
Reticulitermes-3    KFPFRPRSIANKFKNRSLRRQ-KRTSGGVADECCINKGCTIGELREYCAER*--------
Coptotermes-1       KFPFRSRSVANTFKNRSLRRL-KRNSGGVADECCINKGCTLGELREYCAER*--------
Coptotermes-2       KFPFYPRSAANKFKKRSFRRQ-KR-EGGVADECCINKGCTVTELRQYCAER*--------
Coptotermes-3       KFPFRPRSVANKFS-RSLRRQ-KRNSRGVADECCINKGCTLRELREYCAQR*--------
Coptotermes-4       ---------ANKLKKRSLRRQ-KR-EGGVADECCINKSCTVTELRQYCAER*--------
Macroteremes-1      MYPFRPRSVANAFTNRSLRRQ-RRTPRGVADECCIYKGCTVSELRAYCAER*--------
Macroteremes-2      KYPFHSRSVANTFTNRSLRRQ-KRNDGDVANECCIYKGCTVSELRAYCGER*--------
Labritermes-1       KYPFRPRSLANMFKNRSLRRE-KRNDGGVADECCIKKGCIISELRAYCAER*--------
Labritermes-2       KYPFRPRSLANMFKNRSLRRQ-KRTEGGVADECCIYKGCTYSELRAYCAER*--------
                    121.....130.......140.......150.......160.......170........
```

Figure S7. Sequence alignment of the predicted brovirpins from two cockroach species, *Periplaneta americana* and *Cryptocercus wrighti* and ten termite species, *Mastotermes darwiniensis, Zootermopis nevadensis, Hodotermopsis sjostedti, Cryptotermes secundus, Prorhinotermes simplex, Coptotermes formosanus, Reticulitermes speratus, Macrotermes natalensis, Labritermes buttelreepeni* and *Nasutitermes takasagoensis.* Note that the coding sequence for brovipirn 4 from *Coptotermes* contains as stop codon (highlighted in yellow) and is thus unlikely to be functional and that some sequences are incomplete due to lack of data. Other highlighting of residues as in figure S1.

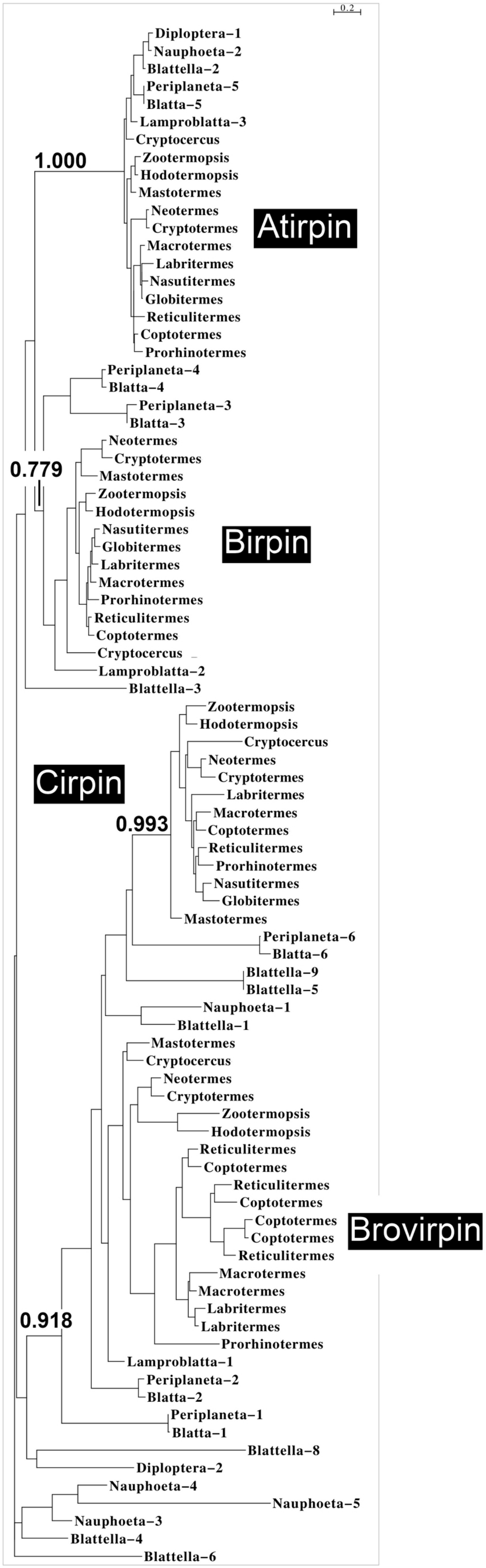

Figure S8. Sequence similarity tree of Blattodea sirps.

```
M.nat-aa                        ValAlaAspLeuLeuMetAlaIleTyrLeuLeuValThrGly
M.nat-nt gtgataataatgctgttagttgcagacctattaatggcaatctatctactggtgactgga
C.sec-nt ataatattggtttccgcagtttcagatttgttaatggcgttttatctactggtgattggg
C.sec-aa                        ValSerAspLeuLeuMetAlaPheTyrLeuLeuValIleGly

M.nat-aa ThrGlnGlyCys***PheArgGlyHisTyrHisArgAspAlaHisSerTrpIleSerSer
M.nat-nt actcagggttgttaattccg-ggacattatcacagagaggcacacagctggatatcgtct
C.sec-nt atccaggactgtcagttccgaggcaactatcacaaggaggctcacaagtggatgtcatct
C.sec-aa IleGlnAspCysGlnPheArgGlyAsnTyrHisLysAspAlaHisLysTrpMetSerSer

M.nat-aa ***ValCysThrLeuIleGlyMetValAlaIleThrSerSerAsp
M.nat-nt tgagtatgcacgctaattggcatggtcgcgatcacgtcatcagaagtgaatatcaaatac
C.sec-nt tggggatgcacgctcatcggtatggtcgcaatgacgtcatcagaagtgagtctcagcctc
C.sec-aa TrpGlyCysThrLeuIleGlyMetValAlaMetThrSerSerAsp
```

Figure S9. The only remaining "coding" exon of LGR3 from *Macrotermes natalensis*. Nucleotide and conceptual translated amino acid sequences are compared with those from the orthologous exon from *Cryptotermes secundus,* in which LGR3 is presumed to be functional. Highlighted in black is the predicted amino acid sequence encoded by the *C. secundus* exon as well as those amino acid residues that are conserved in *M. natalensis*. Although some amino acid substitutions are conservative, there are also two in-frame stop codons as well as a single nucleotide deletion (all highlighted in yellow). Hence this can not be part of a functional protein. *M.nat-aa* and *M.nat-nt M.natalensis* amino acid and nucleotide sequences respectively, *C.sec-aa* and *C.sec-nt*, the same for *C. secundus*.

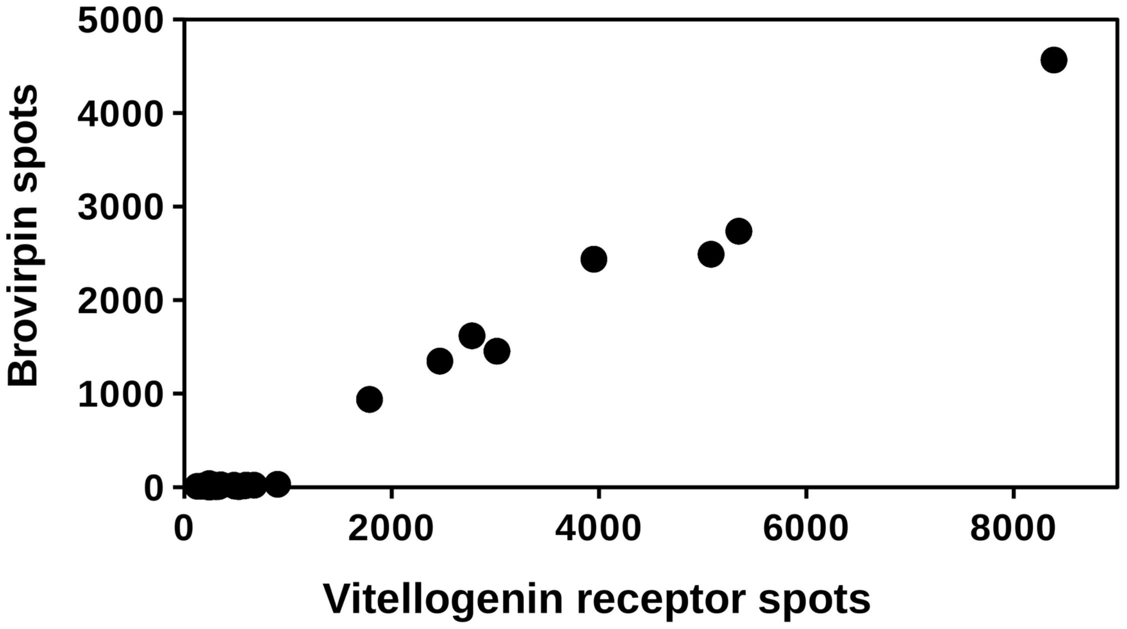

Figure S10. Correlation between the numbers of spots for vitellogenin receptor and brovirpin in transcriptome SRAs from *C. secundus* queens. Note that only those SRAs that have large numbers of brovirpin spots also have large numbers of vitellogenin receptor spots. The SRAs used here are ERR2615943, ERR2615944, ERR2615945, ERR2615946, SRR5457742, SRR5457743, SRR5457744, SRR5457745, SRR12381714, SRR12381716, SRR12381718, SRR12381721, SRR12381723, SRR12381725, SRR12381727, SRR12381728, SRR12381730, SRR12381732, SRR12381734, SRR12381736, SRR12381738, SRR12381740, SRR12381743, SRR12381744, SRR12381745, SRR12381746, SRR12381747, SRR12381748, SRR12381755 and SRR12381756.

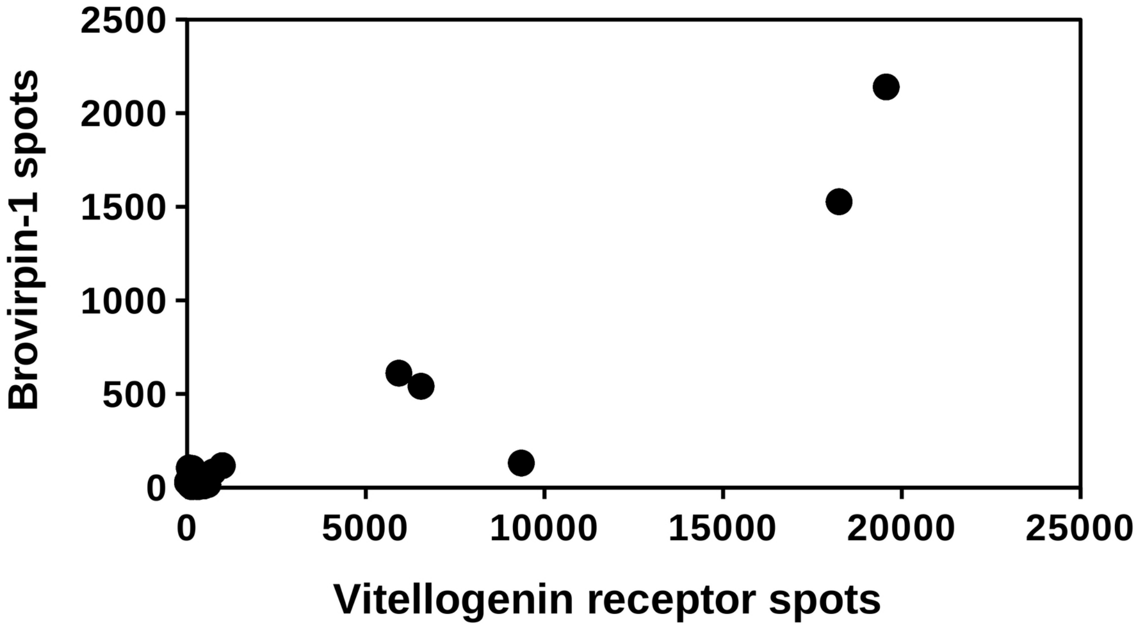

Figure S11. Correlation between the numbers of spots for vitellogenin receptor and brovirpin in transcriptome SRAs from *M. natalensis*. Note that only those SRAs that have large numbers of brovirpin spots also have large numbers of vitellogenin receptor spots. The SRAs used here are : SRR13396076, SRR13396077, SRR13396078, SRR13396079, SRR13396080, SRR13396081, SRR13396082, SRR13396083, SRR13396084, SRR13396085, SRR13396086, SRR13396087, SRR13396088, SRR13396089, SRR13396090, SRR13396091, SRR13396092, SRR13396093, SRR13396094, SRR13396095, SRR13396096, SRR13396097, SRR13396098, SRR13396099 and SRR13396100.

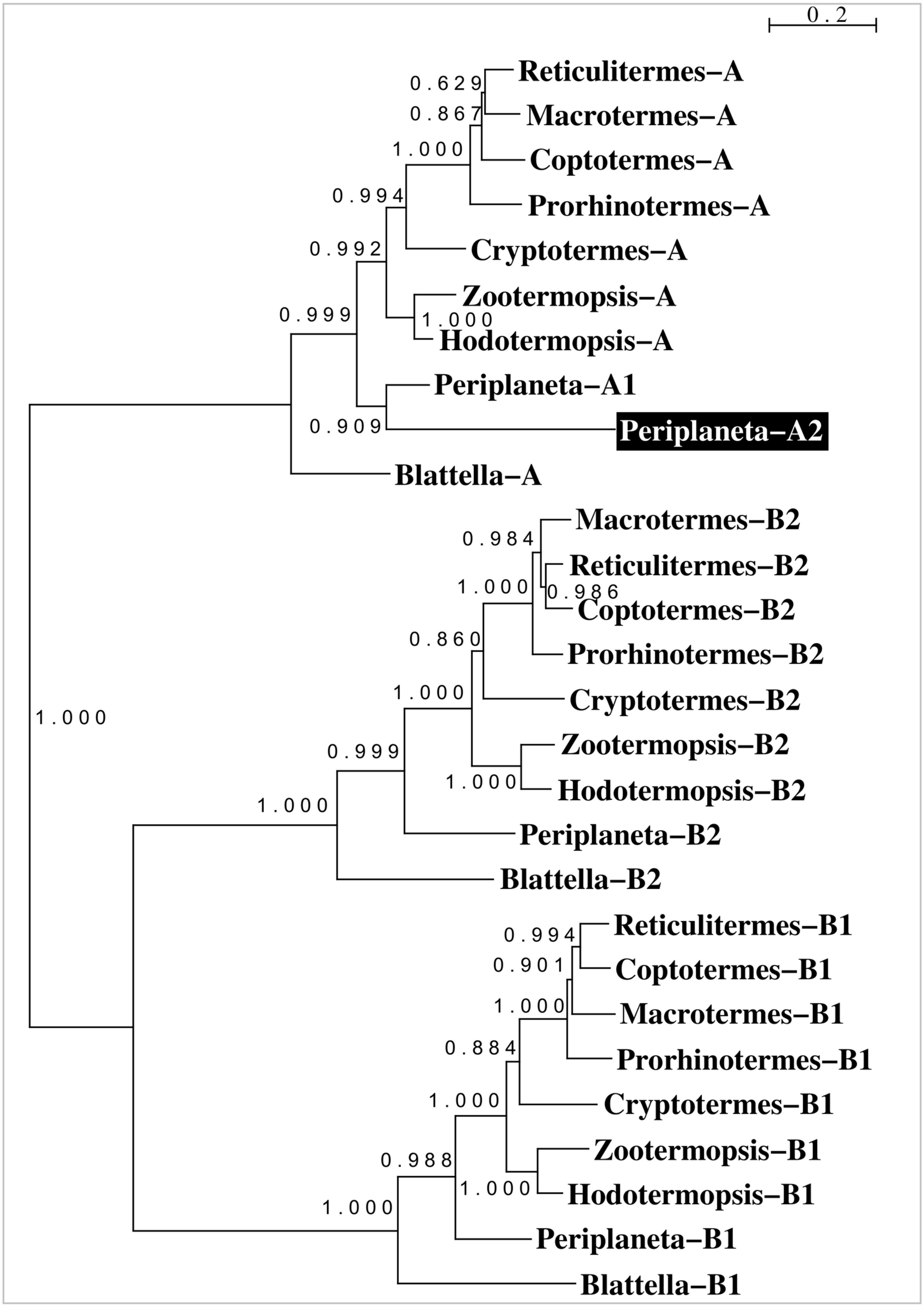

Figure S12. Phylogenetic tree of the termite insulin RTKs, showing that although *Periplaneta* has four such receptors, termites have only three.

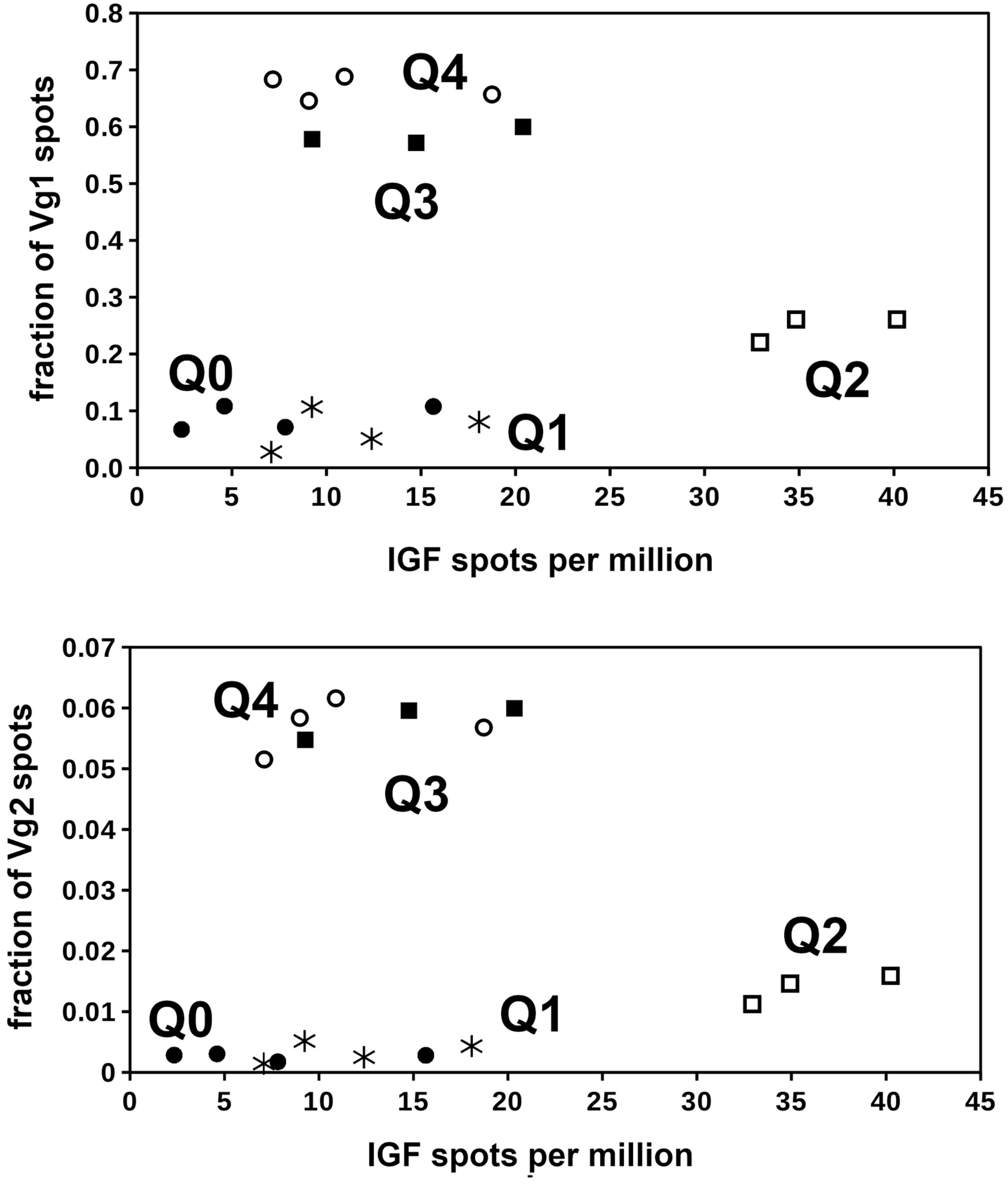

Figure S13. Top panel shows the correlation between the number of IGF and vitellogenin 1 spots in *M. natalensis* queens of different ages, Q0 (closed circles), Q1 (asterisks), Q2 (open squares), Q3 (closed squares) and Q4 (open circles). Note that in Q0 and Q1 queens the fraction of vitellogenin 1 spots is similar, that it increases significantly in Q2 queens and then becomes very high in Q3 and Q4 queens. The bottom panel show the correlation between IGF reads and vitellogenin 2, thus illustrating that qualitatively the results are identical for vitellogenins 1 and 2. Note that IGF expression appears highest in Q2 queens.

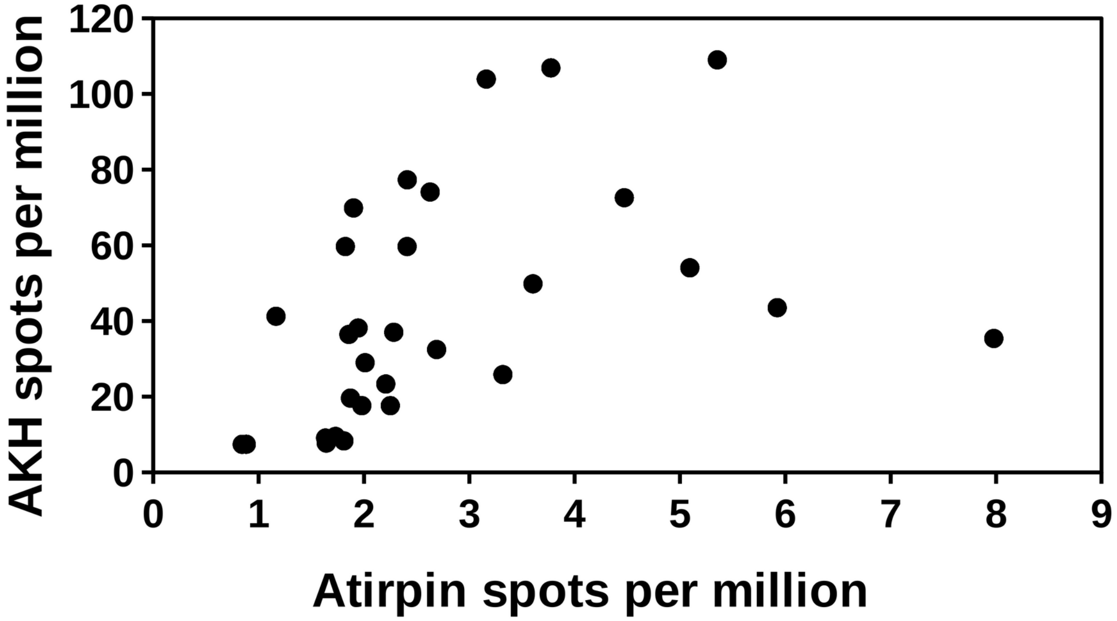

Figure S14. Data from *C. secundus* queens showing the correlation between spots for atirpin and AKH. Same data those in figs. 7-9 from the main text.
